# Collimation and Exposure Parameter Influence Image Quality and Potential Radiation Dose to the Eye Lens of Personnel in Computed Radiography of the Canine Pelvis

**DOI:** 10.3389/fvets.2021.684064

**Published:** 2021-12-14

**Authors:** Malene Bisgaard, Fintan J. McEvoy, Dorte Hald Nielsen, Clara Allberg, Anna V. Müller, Signe Timm, Signe N. Meyer, Line Marie Johansen, Stine Pedersen, Helle Precht

**Affiliations:** ^1^Department of Radiology, Lillebaelt Hospital, University Hospitals of Southern Denmark, Kolding, Denmark; ^2^Health Sciences Research Centre, UCL University College, Odense, Denmark; ^3^Department of Veterinary Clinical Sciences, Faculty of Health and Medical Sciences, University of Copenhagen, Frederiksberg, Denmark; ^4^Department of Regional Health Research, University of Southern Denmark, Kolding, Denmark; ^5^Lillebaelt Hospital, University Hospitals of Southern Denmark, Kolding, Denmark

**Keywords:** collimation field, exposure parameters, image quality, radiation safety, computed radiography, canine pelvis, optimization

## Abstract

**Introduction:** The purpose of this study was to evaluate the effect of collimation on image quality and radiation dose to the eye lenses of the personnel involved in computed radiography of the canine pelvis.

**Materials and Methods:** A retrospective study of canine pelvic radiographs (*N* = 54) was undertaken to evaluate the relationship between image quality and the degree of field the collimation used. This was followed by a prospective cadaver study (*N* = 18) that assessed the effects on image quality and on scattered radiation dose of different collimation field areas and exposure parameters. All radiographs were analyzed for image quality using a Visual Grading Analysis (VGA) with three observers. Finally, the potential scattered radiation dose to the eye lens of personnel restraining a dog for pelvic radiographs was measured.

**Results:** The retrospective study showed a slightly better (statistically non-significant) VGA score for the radiographs with optimal collimation. Spatial and contrast resolution and image sharpness showed the greatest improvement in response to minimizing the collimation field. The prospective study showed slightly better VGA scores (improved image quality) with the optimal collimation. Increasing the exposure factors especially the tube current and exposure time (mAs) resulted in improved low contrast resolution and less noise in the radiographs. The potential eye lens radiation dose increased by 14, 28, and 40% [default exposures, increased the tube peak potential (kVp), increased mAs, respectively] as a result of reduced collimation (increased beam size).

**Conclusion:** The degree of collimation has no statistically significant on image quality in canine pelvic radiology for the range of collimation used but does have an impact on potential radiation dose to personnel in the x-ray room. With regard to radiation safety, increases in kVp are associated with less potential scatter radiation exposure compared to comparable increases in mAs.

## Introduction

Radiology-based imaging diagnosis is constantly evolving, and it remains a valuable tool in diagnosis and clinical decision-making in veterinary medicine. The use of conventional radiography increases every year and the majority of veterinary clinics in many countries perform x-ray examinations daily (1). High image quality radiographs are required for clinical diagnosis.

Image quality is a collective term, which covers various factors that individually affect visualization of anatomical structures in the radiograph (1–3). Important parameters for image quality are spatial resolution (i.e., visualization of small details), contrast resolution (i.e., discrimination of structures with different radiopacities), sharpness, and homogeneity. Image noise including quantum mottle will appear if insufficient x-ray photons are used for the exposure (2, 3). An acceptable radiograph can be obtained if the animal lies still and if the following parameters are optimized: (a) exposure parameters (including exposure time), (b) focal spot to detector and object-to-detector distances, and (c) the beam collimation. All parameters mentioned will influence the radiograph and thus the process of making the diagnosis (4).

Scattered radiation is secondary radiation that occurs when the x-ray beam interacts with material, and is emitted in all directions from patients during radiography. The amount of scattered radiation produced can be reduced by collimation, decreased tube peak potential (kVp), decreased tube current and exposure time (mAs), external filtration in the x-ray tube, and compression. The amount of scattered radiation reaching personnel can be reduced by increasing the distance between the patient and personnel and by the use of lead shielding (1, 4, 5). The amount of scatter reaching the detector can be reduced by the use of an anti-scatter grid (5). Scattered radiation has a negative effect on contrast resolution and results in radiation dose to both the patient and the personnel present in the room at the time of radiography (1, 6). In most jurisdictions, the “As Low As Reasonable Achievable” (ALARA) principle must be applied during veterinary radiography. This is particularly important in regions where it is considered reasonable for personnel to be present in the room during certain x-ray exposures, to restrain and position the animal. Thousands of pelvic radiographs are acquired yearly for hip dysplasia screening (6) where positioning is certainly important. In many countries, personnel carefully position the dog during the examination. Additional personnel may be required in the radiography room to attend to other practical issues related to general anesthesia or sedation (4). In such situations, the veterinary personnel will be exposed to scatter radiation as a result of their work activity, thus increasing the risk of developing cataract (7). Therefore, it is important and a legal requirement to keep the radiation doses to personnel both below limits and in accordance with ALARA principles (8, 9).

The aims of this study were to evaluate the effect of collimation and different exposure parameters on image quality and to measure the radiation dose to the eye lenses of the restrainer involved in computed radiography of the canine pelvis.

## Materials and Methods

This study comprised three parts. The first part examined the degree of collimation, the visual image quality, and the intra- and inter-observer agreement of 54 retrospective radiographs of the canine pelvis. The second part compared the image quality of two different collimation fields and exposure parameters of the same dog cadaver. The third part of the study measured the scattered radiation at the level of potential eye lenses of a simulated person restraining a dog cadaver for pelvic radiograph.

All procedures were performed in accordance with local and European data protection regulations. The ethics committee in the Region of Southern Denmark waived its requirement for ethical approval for the study.

### Radiography Equipment

All included radiographs were acquired with a Sedecal x-ray system (high frequency x-ray Generator, Model no. SHF-330, serial no. G-29078; Madrid, Spain) with a fixed source-to-image distance (SID) of 100 cm. The x-ray table was equipped with a linear focused grid: 40 lp/mm, ratio 8:1, SID: 100 cm (Dunlee Medical Components, Best, The Netherlands). All radiographs were produced using an AFGA Computed Radiography (CR) system (CR MD4.0T General; Gent, Belgium) with a high-resolution digital cassette; size 43^*^35 cm of 10 pixel/mm and an AGFA CR 30-C reader.

### Retrospective Collimation and Image Quality Assessment

A total of 54 of the most recently acquired canine pelvic standard ventrodorsal (VD) projection radiographs were collected retrospectively from a Danish Veterinary Hospital. The body weight of the dogs and the exposure parameters were not available. All radiographs were anonymized, exported in dicom (dcm) file format and assigned a random identity number for the later evaluation of image quality.

**Figure 1:**
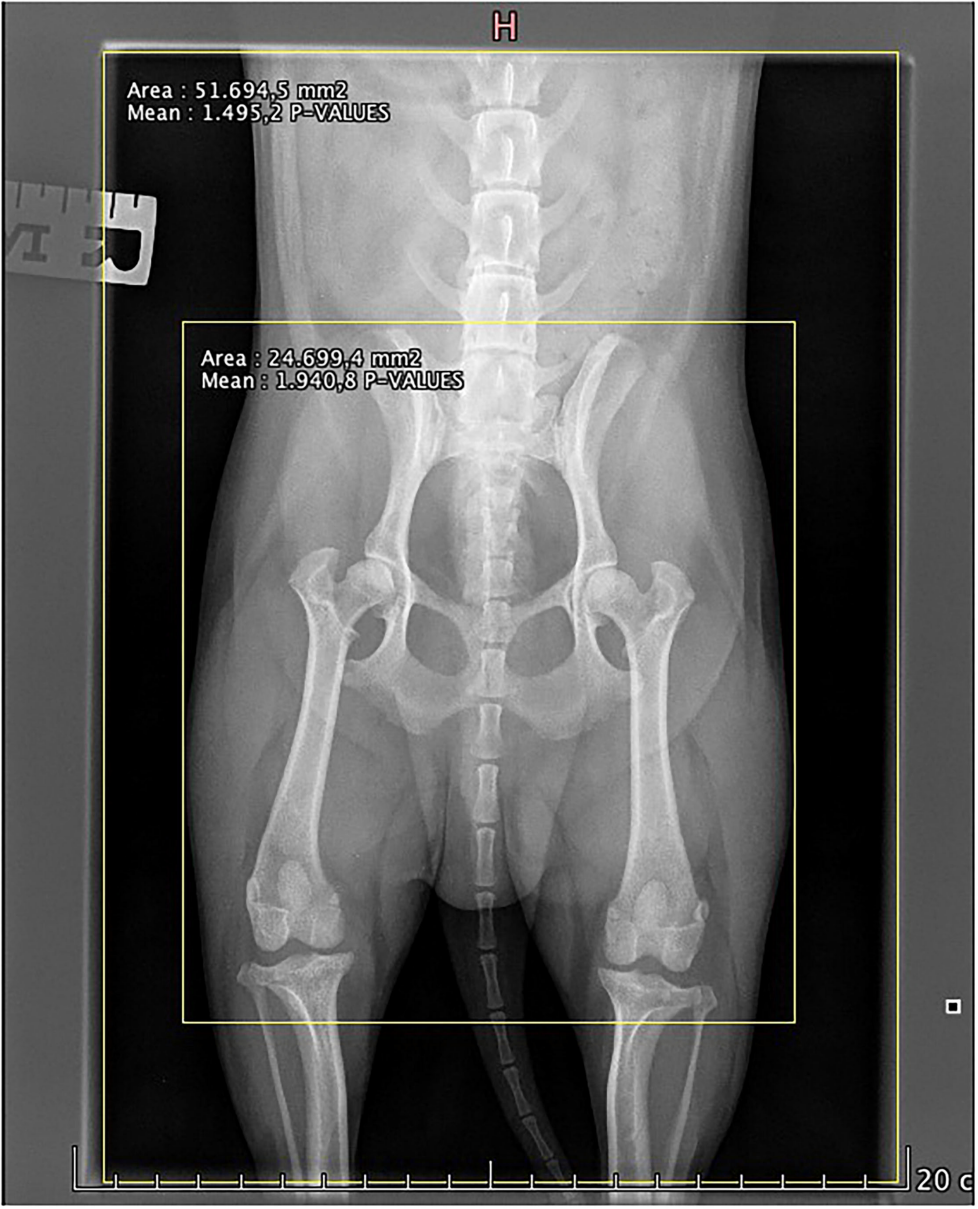
Example on how the collimation measurements were calculated. The area of the large yellow box shows the actual collimation as area of the small yellow box shows the optimal collimation. The classifications—small: well-collimated, medium: fairly collimated, and large: poorly collimated—were calculated on the absolute difference in area between these two measurements, i.e., Actual collimation (mm^2^)—optimal collimation (mm^2^), using the ranges 0–1,800 mm^2^, 1,800–2,700 mm^2^, and 2,700–4,400 mm^2^, respectively.

To evaluate the collimation of the retrospective images, an established method was used to quantify the degree of collimation present in order to calculate the deviation (if any) between the current and optimal collimation (10), as shown in Figure 1. The radiographs were then divided into three groups, according to degree of difference between the actual and optimal collimation.

### Prospective Collimation and Image Quality Assessment

**Figure 2:**
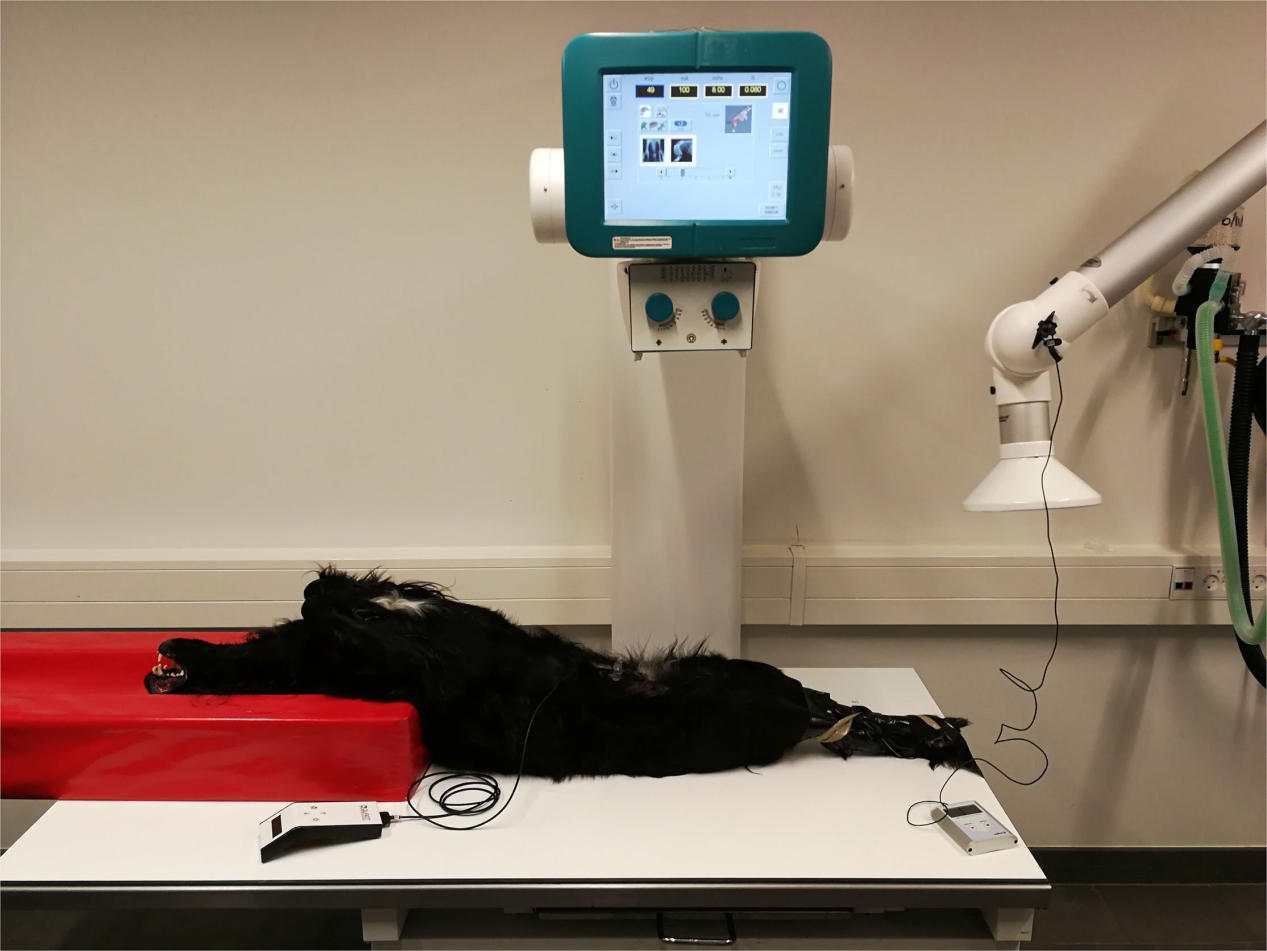
The prospective study setup including the radiation dose measurements for scattered radiation to potential eye lens. The dog was in dorsal recumbency with the head to the left supported by a foam cushion. Hind limbs were fixed with tape. The dosimeter was placed 44 cm from the dog simulating the location of the eyes of the restrainer.

To examine the effects of collimation and different exposure parameters on image quality, and to evaluate a predicted dose to eye lens of staff present during radiography, a canine cadaver and a mechanical stand were used to avoid unnecessary exposure to radiation, pain, anxiety, or stress in dogs and humans (11). The cadaver was a Labrador/Spaniel mixed breed dog: 55 cm height and 22 kg. A written consent form was signed by the owner before inclusion of the dog (12). The cadaver was positioned as best as possible for a VD projection. The chest was placed in a support cushion and the pelvic limbs were extended caudally, rotated inwards, and held in place with tape, as shown in Figure 2. The pelvic height was 10 cm.

**Figure 3:**
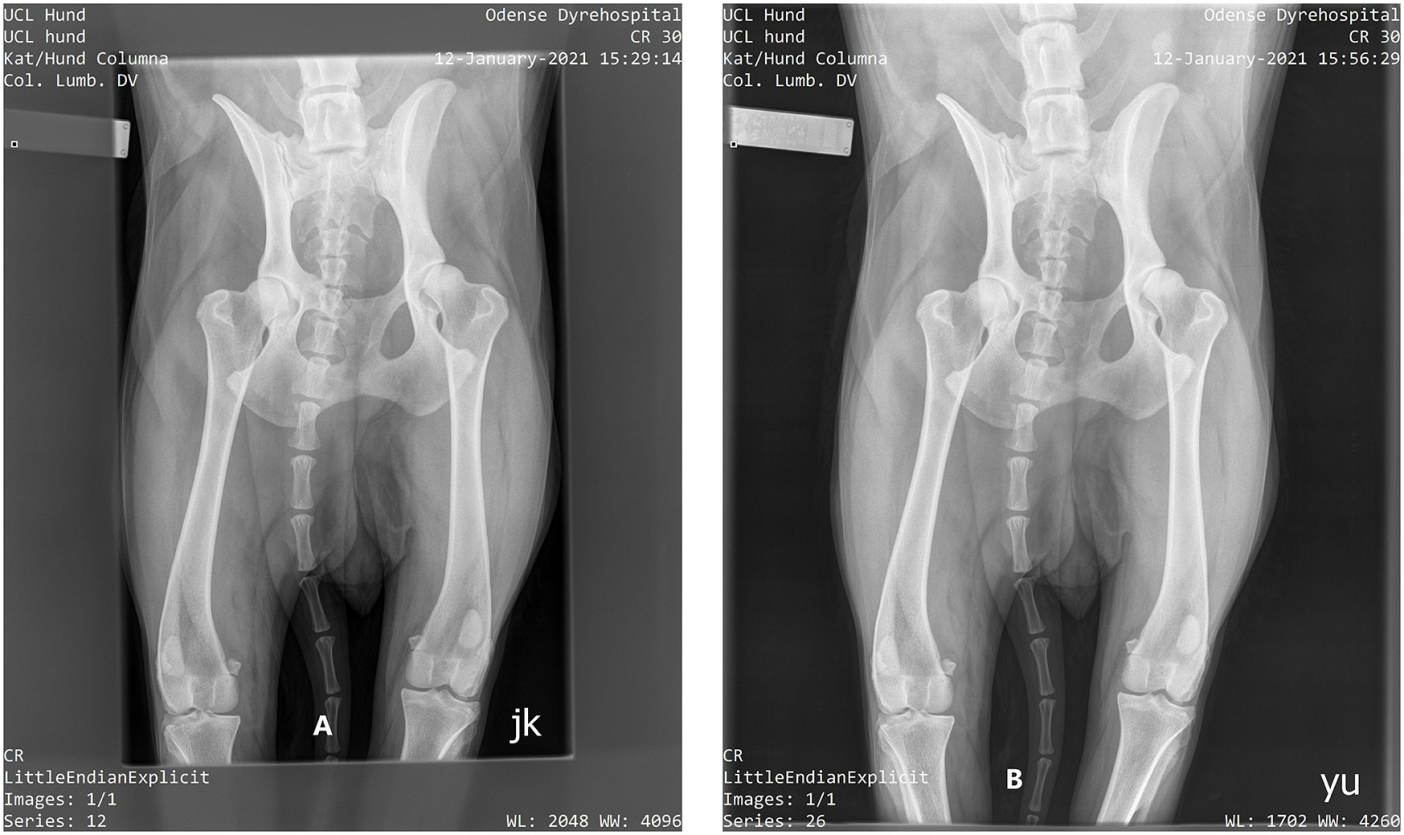
Radiograph of dog cadaver. **(A)** (left of figure): optimal collimation. **(B)** Extended collimation.

Two collimation sizes were used. For this particular patient, optimal collimation was set at 31 × 22 cm. Then, a larger field collimation was set at 38.5 × 32 cm (Figure 3). The SID was fixed at 100 cm and only the smaller of the two available focal spot sizes was used. Exposure parameters used are shown in Table 1; default values were those in daily use at the Veterinary Hospital for this type of examination in this patient size. The settings “increased kVp” and “increased mAs” were based on the 15% rule, which states that increasing the kV by 15% has the same effect as doubling the mAs to achieve comparable optical density or noise level reduction in the images (2). Both of the setting “increased kVp” and “increased mAs” should represent a doubling of the default exposure (see Table 1).

**Table 1:** Technical settings used in the prospective study.

	**Optimal collimation**		**Large field collimation**	
	**kVp**	**mAs**	**kVp**	**mAs**
Default	49	8	49	8
Increased kVp	55	8	55	8
Increased mAs	49	16	49	16

### Visual Image Quality Evaluation

Image quality was subjectively evaluated by absolute Visual Grading Analysis (VGA) in both the retrospective and the prospective study. VGA is a well-documented grading method (2, 13). Absolute VGA is based on image criteria describing the visualization of five anatomical structures; see Table 2 for the criteria used in this study. The radiographs were viewed individually, without any references for comparison. The VGA scores were awarded on a five-point scale, with scores of 1, 2, 3, 4, and 5 to indicate the visibility of the structure as being not, poorly, moderately, adequately and very well-reproduced, respectively. The mean of the five individual image criteria scores was determined for each image for each reader, and the mean of these reader VGA scores was taken as the overall VGA score (VGAS) for each image. The score for each image is thus a mean of means. The minimum score an image could receive was 1 and the maximum was 5.

**Table 2:** Image criteria used in the VGA related to definition for the observers and technical image quality used in the Discussion section.

**Question**	**Image criteria**	**Definition**	**Technical image quality**
1	Sharpness of trabecular pattern in left femur	The structure is clearly defined and seen sharply	Spatial resolution
2	Visualization of the demarcation between compact bone and spongy bone in left diaphysis of femur	The transition is well-defined	Low contrast resolution
3	Homogeneity in soft tissue next to right coxae	The area is seen with a uniform gray tone	Noise
4	Sharp representation of right acetabulum	The anterior and posterior part of acetabulum is well-defined	Sharpness and contrast resolution
5	Visualization of right patella	The structures are clearly depicted, not necessarily in detail, but visible.	Low contrast resolution

All radiographs were scored digitally in a program: “Viewer for Digital Evaluation of X-ray images” (ViewDEX) (ViewDEX 2.48, Västra Götalandsregionen, Sweden) (14). Two sessions (retrospective- and prospective study) were performed including the same three veterinary radiologists with 4 (CA), 35 (FJM), and 43 (DHN) years of experience in veterinary diagnostic imaging, respectively. ViewDEX allowed each image to be visualized on the same diagnostic DICOM monitor individually in a random order. All observers were given unlimited time and worked undisturbed. Responses were automatically logged in data files created by the program and were later exported to a spreadsheet (Excel, Microsoft, Salt Lake City, UT, USA) format.

The total number of radiographs for the retrospective study was 60 (54 + 6 repeated) and that for the prospective study was 23 (18 + 5 repeated). The repeated images were included to validate the data as a measure of intra-observer agreement; this validation should be performed on 10% of all images or using a minimum of five radiographs (13).

### Quantified Dose Measurements and Potential Eye Lens Doses in Prospective Study

Also using the cadaver, the radiation dose was measured as entrance skin dose and potential dose to the eye lens of personnel present in the room next to the patient during radiography.

**Figure 4:**
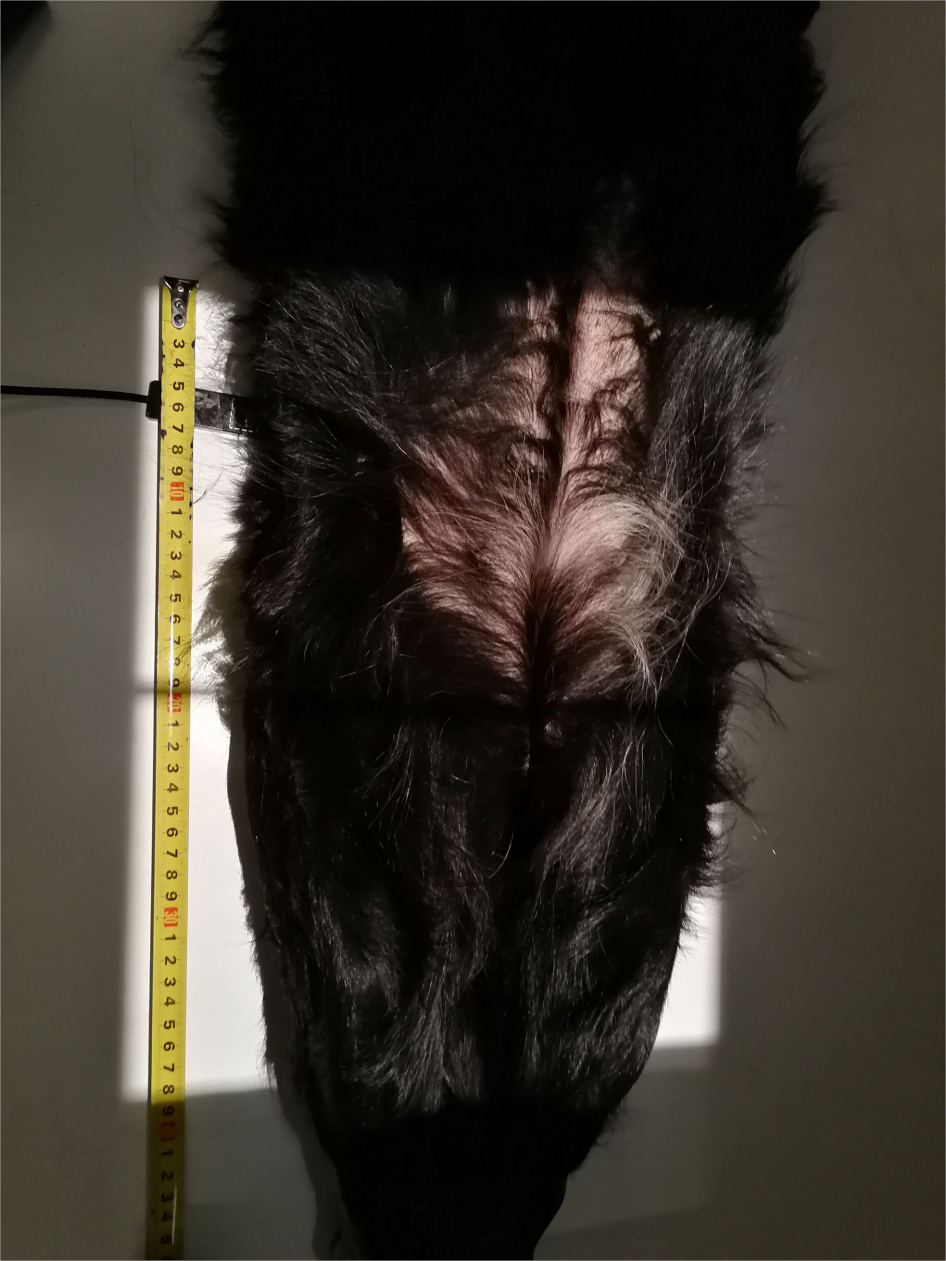
The prospective study setup. The dosimeter (placed to measure entrance skin dose) and its cable can be seen at the top left of the image.

A Quart Dosimeter (QUART didoEASY MR; QUART, Köln, Germany) was used to examine the x-ray output of the generator and tube as the entrance skin dose (ESD). ESD is the measure of the radiation dose from the primary beam that reach the detector (2). The dosimeter was placed in the direct beam close to the collimation edge, during the study as shown in Figure 4.

A Direct Dosimeter (Unfors EDD-30; RaySafe, Billdal; Sweden) was used to evaluate the potential eye lens dose given by scattered radiation for different degrees of collimation and for different exposure values. The dosimeter was fixed on a mechanical stand positioned at a height corresponding to the eyes of personnel restraining and positioning the dog for a pelvic radiograph. The distance from the point where the central x-ray beam hit the cadaver to the dosimeter was 86 cm (Figure 2). Ten exposures and measurements were made to secure against possible statistical variations in the dosimeter and beam. After each exposure, the measured scatter radiation was noted and the mean of the 10 repetition measurements was used.

### Statistical Analyses

For the retrospective study, a Kruskal–Wallis rank sum test was used to test the associations between image quality scores and collimation sizes. In the prospective study, Wilcoxon rank sum test was used to test the association between different degrees of collimation and exposure parameters on simulated eye lens doses, respectively.

Intra- and inter-observer agreement was reported as mean ICC estimated in random effects models (15). The ICCs were interpreted as follows: 0–0.50 poor, 0.51–0.75 moderate, 0.76–0.90 good, >0.90 excellent.

All statistical analyses were performed using STATA 16 IC (StataCorp, College Station, TX, USA).

## Results

### Retrospective Collimation and Image Quality Assessment

**Figure 5:**
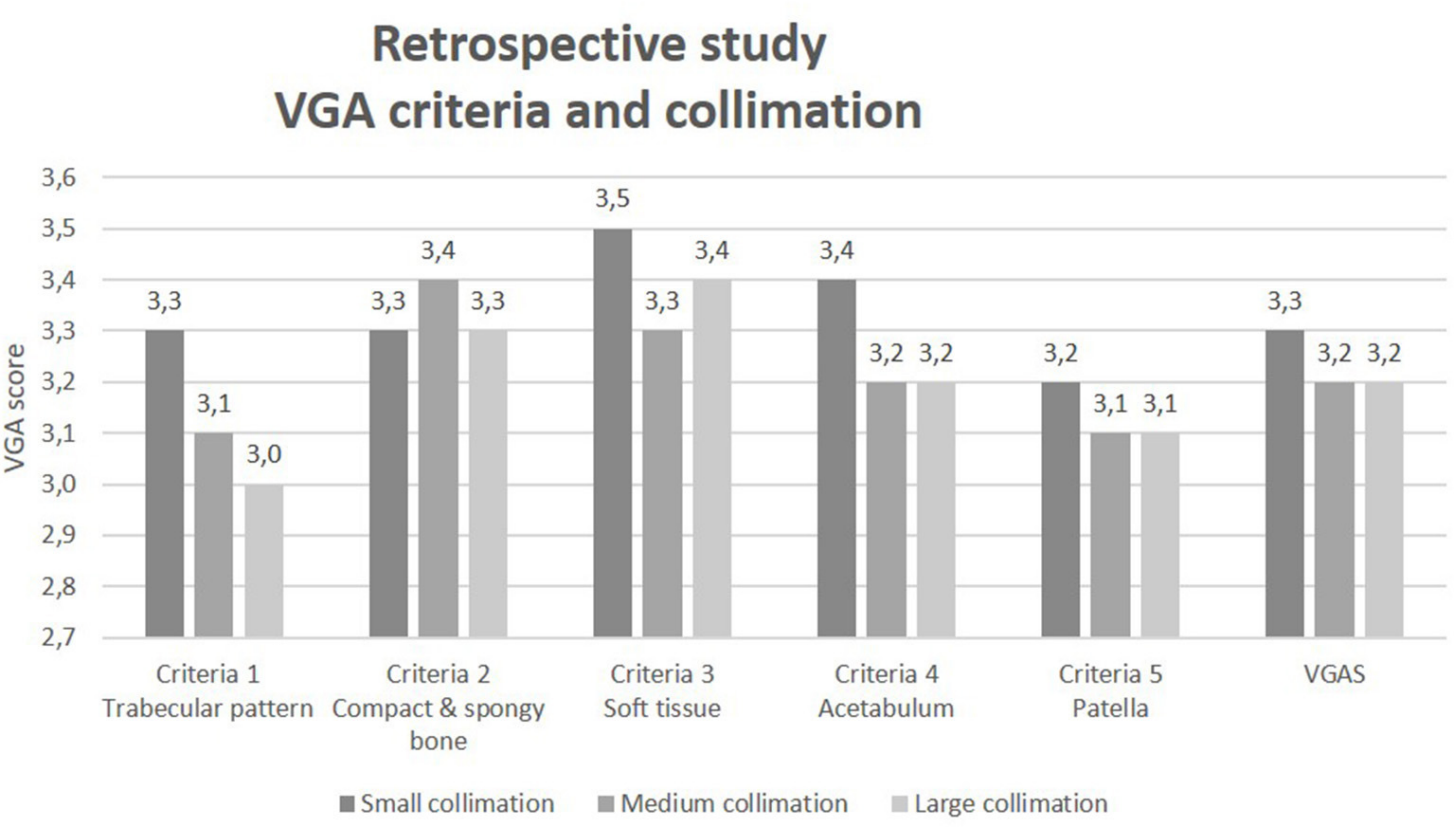
VGA criteria according to collimation groups small, medium, or large.

The absolute difference in area between the actual collimation used the optimal collimation to place all images in one of three groups: (1) small: well-collimated (0–1,800 mm^2^, *n* = 17); (2) medium: fairly collimated (1,801–2,700 mm^2^, *n* = 17), and (3) large: poorly collimated (2,701–4,400 mm^2^, *n* = 20). The results comparing collimation and VGA score for each VGA criterion and VGAS are shown in Figure 5.

A Kruskal–Wallis test was conducted to determine whether the image quality grades (VGA) differed according to three collimation groups: small, medium, and large. It showed no significant difference between the collimation size groups for either the VGAS or the individual VGA image criteria scores (Crit 1–5), see Table 3.

**Table 3:** Kruskal–Wallis test for each collimation size reported as Rank Sum for each collimation and VGA criteria, chi-square, df, and *p*-value.

**Collimation size**	***N***	**Rank sum**					
		**Crit 1**	**Crit 2**	**Crit 3**	**Crit 4**	**Crit 5**	**VGAS**
Small	17	518	409	513	497	501	492
Medium	17	435	484	406	440	448	437
Large	20	533	593	566	549	536	556
Chi-square (df)		2	2	2	2	2	2
*H*		0.948	1.317	1.522	0.389	0.405	0.372
*p*-value		0.62	0.51	0.46	0.82	0.81	0.83

The intra-observer agreements showed an ICC value for observers 1 and 2 of 0.90 and 0.83, respectively. These values indicate a good to excellent agreement. Observer 3 had an ICC value of 0.35 defining a poor agreement. The inter-observer agreement showed a poor agreement based on an ICC value on 0.23.

### Prospective Collimation and Image Quality Assessment

**Figure 6:**
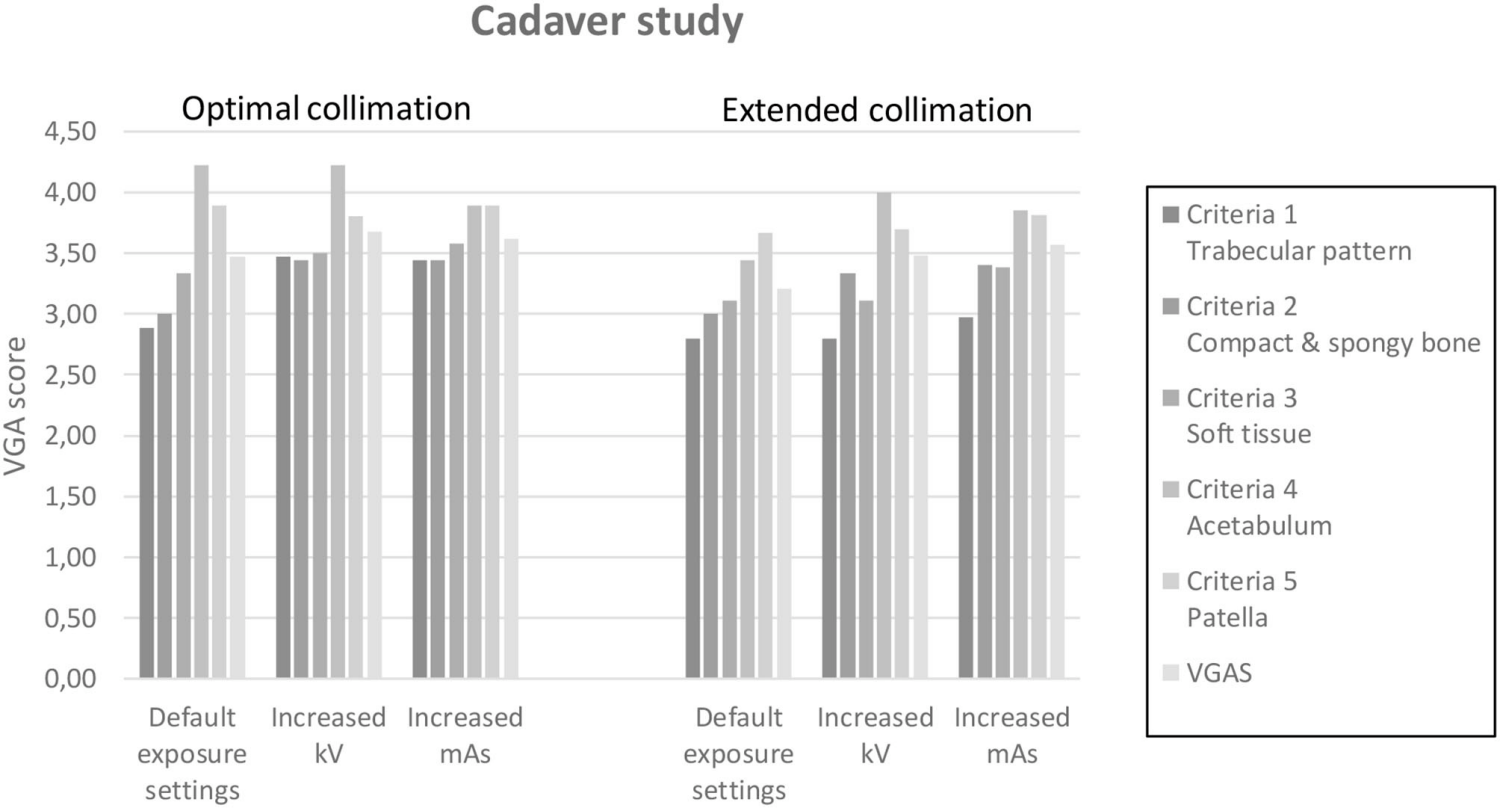
Bar chart divided into two sections with the optimal and extended collimation. Each of these two sections were further divided into three sections: Default exposure settings, increased kV, and increased mAs. All five VGA criteria and VGAS were represented for each column.

Results of optimal and extended collimation according to variant exposure parameters are shown in Figure 6. Small variations in image quality are seen between the two collimation sizes with slightly higher scores for the optimal collimation at all exposure settings.

The intra-observer agreements showed a mean ICC for VGAS of 0.33, indicating a moderate agreement. The inter-observer agreement showed a mean ICC for VGAS of 0.071 reporting a poor agreement.

### Potential Eye Lens Doses in Prospective Study

**Figure 7:**
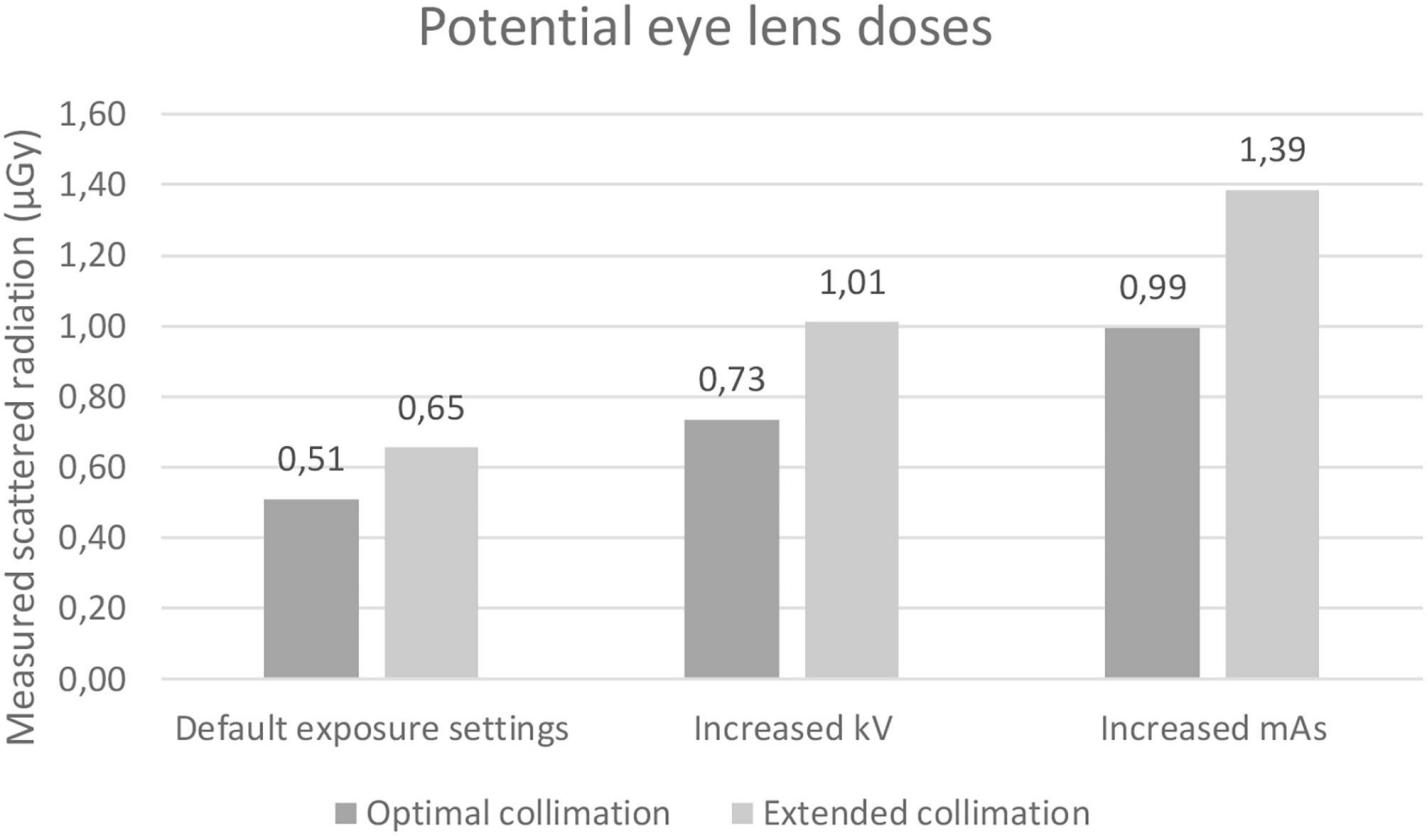
Bar chart with potential eye lens dose for each of the three exposure settings: Default, increased kV, and increased mAs. Each presented with optimal and extended collimation.

Simulated eye lens doses from scattered radiation were measured for different degrees of collimation and exposure parameters. Wilcoxon rank sum test showed statistically significant median eye lens doses in the two exposure groups (8 or 16 mAs, *p* < 0.01) but no difference on median eye lens dose according to collimation (49 or 55 kVp, *p* = 0.39). All results are presented in Figure 7.

## Discussion

This study set out determine if the degree of collimation and exposure parameters used influence image quality and potential radiation dose to the eye lenses of personnel involved in computed radiography of canine pelvis using two separate evaluations: a retrospective study and a prospective study.

### Retrospective Collimation and Image Quality Assessment

The results of the retrospective study evaluating image quality in 54 radiographs of canine pelvis indicate that optimal collimation had a slightly higher VGA score for most VGA criteria (all except criteria 2, see Figure 5) although not statistically significant (*p* = 0.46–0.83). This propensity corresponds with the theory, as the amount of scattered radiation is generally proportional to the total mass of tissue contained within the primary x-ray beam. Increasing the exposed field size increases the total amount of scattered radiation and the value of the scatter contrast-reduction factors. Reducing the size of the scattered radiation source by collimation is an effective way of improving image quality (contrast resolution) and reducing the amount of scattered radiation around the patient (16, 17). The pelvis is one of the larger anatomical areas in dogs and radiographs of the pelvis include considerable regions of scatter generating soft tissues such as muscles. The size of the 54 included pelvis images was unknown. Different dog breeds vary in size, which will influence the VGA results in our study. With the use of a physical grid for pelvic examinations, most of the scattered radiation influencing the image quality will be removed, which could partly explain why the differences in the VGA are non-significant. A future study should investigate the effect when using a grid. The Kruskal–Wallis test showed no significant difference for any VGA criteria between the small (optimal), medium, and large (extended) collimation sizes with a Rank Sum for VGAS varying from 492, 437, to 556 for small, medium, and large collimation sizes, respectively. The spread (*H* value) of data in the included VGA criteria showed the highest for criteria 3 and 2 (1.522 and 1.317) and the lowest for criteria 4 and 5 (0.389 and 0.405). The degree of freedom (df) was 2 for all criteria and VGAS included, indicating that the amount of data for the analyses is limited as more consistent evaluators could have been included. These results can be compared with those presented in a PhD thesis by Koernig (18). He found that image quality for the VD images was significantly better in the collimated approach compared to a whole-body radiograph with over twice as much scatter radiation detected during the whole-body images compared to the collimated images (18).

Each VGA criterion corresponds to different factors in image quality (see Table 2). Criteria 1, 4, and 5 all showed a higher VGA score for the optimal collimation compared to the medium and large collimation field in groups. Criterion 1 corresponds to spatial resolution based on the trabecular pattern, which increased with a smaller collimation field. Less scattered radiation could hereby increase the visualized details with high contrast differences. Criteria 4 and 5 both correspond to contrast resolution, which in theory is the image quality factor most prejudiced by scattered radiation. Again, a smaller collimation field corresponds with a slight increase in the VGA score. This was seen in a study by Precht et al. (19), showing that the scatter radiation had a negative impact on the image contrast because the scatter radiation strikes the image plate without structural tissue information (19). In this study, however, this relationship between collimation and image quality is not seen to a statistically significant degree. VGA criteria 2 focuses on the visualized difference between compact and cancellous bone structure and indicates low contrast resolution. It is the only VGA criterion that did not improve in mean VGA score with reducing collimation size. This finding and also the lack of statistically significant improvements in image quality with collimation for the other criteria, could be explained by considering that some of the collimation applied to the beam simply excluded air space around the patient (which does not generate scatter) or excluded less bulky (relative to the pelvis) body parts such as the limb distal to the stifle (which generate relatively little scatter). Therefore, the image quality benefits of collimation may be more obvious with smaller regions of interest, for example, the canine shoulder or elbow, where larger differences between optimal and extended collimation are possible. It is well-accepted that collimation size can influence image quality as described and demonstrated by Karami et al. (20) and Pazanin et al. (21) who concluded that optimization of collimation resulted in a reduction of the primary collimation field of 40% and in improved image quality, with a significant difference of 24% in 55 patients.

The intra-observer agreement showed a good to excellent agreement for observers 1 (0.90) and 2 (0.83) but a poor agreement for observer 3 (0.35). This may be due to the observers' different experiences and perceptions of the radiographs as observers 1 and 2 are the most experienced in the field available in Denmark. It should be noted also that these ICC values were based on five repeated images. The inter-observer agreement showed a poor agreement with an ICC of 0.23, which is similar to comparable studies.

### Prospective Collimation and Image Quality Assessment

As presented in Figure 6, the VGA score for nearly all VGA criteria was slightly higher for optimal collimation than extended collimation field. The lowest VGA score was seen for spatial resolution (criteria 1) while the highest score was given to sharpness and contrast resolution in the acetabulum (criteria 4). In theory, optimal collimation should influence contrast resolution the most (5), which also was seen in our result for default and increased kVp settings. This corresponds to a study by Meisinger et al. (16). Increasing mAs did result in increased homogeneity of soft tissue (criteria 3), meaning that less image noise is visualized in the radiographs. Also, the two criteria that focused on low contrast resolution (criteria 2 and 5) did show higher VGA scores for increased mAs images, corresponding to the theory that noise mostly affects low contrast resolution (3), whereas the VGAS and VGA scores for criteria 1 and 4 were higher for the increased kV images compared to increased mAs images. All VGA scores had moderate intra-observer agreement and poor inter-observer agreement. Furthermore, the use of a cadaver did not make it possible to achieve optimal positioning of the pelvis. The suboptimal patient positioning encountered would not have significantly affected the amount of scattered radiation produced.

### Estimated Eye Lens Doses

Scattered radiation was measured at the height of the potential eye lens of simulated personnel restraining a dog. The data on potential eye lens dose showed a statistically significant increase in scattered radiation related to the extended collimation for tube current and exposure time and not only a visual difference for the tube current (default exposure parameters: 0.51–0.65 μGy; increased kVp parameters: 0.73–1.01 μGy; increased mAs parameters: 0.99–1.39 μGy). An increase in scattered radiation of 14, 28, and 40% was found in each group between optimal and extended collimation. The highest amount of scattered radiation was found using an extended collimation with increased mAs (1.39 μGy) and the lowest radiation dose for default exposure parameters and optimal collimation (0.51 μGy). These findings correspond to a study by Meisinger et al. (16). In theory, an increased kVp produces more scatter radiation compared to a corresponding increased mAs (where maintaining optical density in the image is taken as the reference). This is explained by a larger probability of Compton effect with an increased kVp (2). One explanation for our finding that increased mAs resulted in the greatest measured radiation could be that with increasing kVp, the direction of the scatter is more likely to follow the path of the primary beam toward the table with less directed back toward the x-ray tube and dosimeter. Thus, more scatter may have been produced at the increased kVp setting than at the increased mAs setting in general, but less was present in the direction of the tube head where the measurements were made. The exposure parameters we used were increased from default according to the 15% rule used in clinical practice for optimization, stating that increasing the kVp by 15% has the same effect as doubling the mAs receiving comparable signal to the CR plate. Therefore, given our default settings, the kV increase of 10 kVp corresponds to a doubling of the mAs. From a radiation safety aspect, an increase in kV is superior to increased mAs, if more radiation is needed to achieve a good diagnostic radiograph. Since this part of the study was done in a controlled environment to investigate the consequences of parameter changes in relation to eye dose, we would recommend a future study investigating the dose given to the eyes during clinical use, which also includes retakes and analysis of image quality.

### Relation Between the Retrospective and Prospective Study

Some of the radiographs included in our retrospective study had an unnecessarily large collimation. The fact that optimal collimation did not significantly improve the image quality, but did increase the scattered radiation dose to the eyes of the restrainer, is a clear example of dose creep (22), a phenomenon where variations in radiation exposure can occur unnoticed. Our study highlights that dose creep is a real danger in canine pelvic radiography, which has also been described in human pelvic radiography (23). In summary, good radiographic technique minimizes unnecessary radiation dose to the patient and personnel.

Our study only looked at the image quality and eye dose and did not address the possibility to exclude manual restraint for the HD screening program. Santana et al. (24) demonstrated that it might be possible to avoid staff from being exposed while in the room, which could be explored in a future project when evaluating detailed image quality.

### Results Summary

Veterinarians should be aware of the exposure parameters and collimation used during radiography. Collimation size in the context of canine pelvic radiology slightly influences image quality but to levels that may not be detectable. It however clearly has an impact on scattered radiation and on potential dose to personnel in the x-ray room. Unnecessarily high kVp and mAs settings increase the amount of scattered radiation around the patient and conflict with ALARA principles. Unnecessarily high exposures are not associated with an increase in image quality and so provide no diagnostic benefit. Optimal collimation is also associated with a decreased radiation exposure risk. The image quality benefits of collimation that are well-described and well-accepted were present, but only to a marginal degree in this study. More data are warranted to make more robust statistics for canine pelvic radiography, which should be explored in future studies to increase the sensitivity to small image quality benefits that may be and likely are present, but were not detectable in this study.

## Data Availability Statement

The raw data supporting the conclusions of this article will be made available by the authors, without undue reservation.

## Ethics Statement

The animal study was reviewed and approved by Ethical Committee in the Region of Southern Denmark. Written informed consent was obtained from the owners for the participation of their animals in this study.

## Author Contributions

MB: developed methods, supervised in data collection, analyzed data, and wrote the manuscript. FM, DN, and CA: developed methods, supervised in data collection, evaluated images, and commented on the written manuscript. AM: developed methods, supervised in data collection, and contributed intensively to the written manuscript. ST: developed methods, statistical calculations, and commented on the written manuscript. SM, LJ, and SP: developed methods, collected data, set up the data for analyses, and wrote the manuscript. HP: responsible for the entire project, developed methods, supervised in data collection, analyzed data, and contributed to writing the manuscript as commented on the paper. All authors contributed to the article and approved the submitted version.

## Funding

The Danish Veterinary Nurses' Union has financially supported the release/project with 2000 DKR. The Federation of Independent Service Unions has financially supported the release/project with 6500 DKR. The Danish Council of radiographers has financially supported the release/project with 6000 DKR. Research Council, Lillebaelt Hospital, University Hospital of Southern Denmark has financially supported the release/project with 7500 DKR.

## Conflict of Interest

The authors declare that the research was conducted in the absence of any commercial or financial relationships that could be construed as a potential conflict of interest.

## Publisher's Note

All claims expressed in this article are solely those of the authors and do not necessarily represent those of their affiliated organizations, or those of the publisher, the editors and the reviewers. Any product that may be evaluated in this article, or claim that may be made by its manufacturer, is not guaranteed or endorsed by the publisher.

## References

[1] Barber J McNulty JP. Investigation into scatter radiation dose levels received by a restrainer in small animal radiography. J Small Anim Pract. (2012) 53:578–85. 10.1111/j.1748-5827.2012.01257.x 22861077

[2] Seeram E Davidson R Bushong S Swan H. Image quality assessment tools for radiation dose optimization in digital radiography: an overview. Radiol Technol. (2014) 85:555–62. 24806056

[3] Huda W Abrahams RB. X-ray-based medical imaging and resolution. AJR Am J Roentgenol. (2015) 204:W393–7. 10.2214/AJR.14.13126 25794088

[4] Mayer MN Koehncke NK Belotta AF Cheveldae IT Waldner CL. Use of personal protective equipment in a radiology room at a veterinary teaching hospital. Vet Radiol Ultrasound. (2018) 59:137–46. 10.1111/vru.12583 29230889

[5] Bushong SC. Radiologic Science for Technologists: Physics, Biology, and Protection. St. Louis, MO: Elsevier (2013). p. 665.

[6] Moorman L Precht H Jensen J Svalastoga E Nielsen DH Proschowsky HF. Assessment of image quality in digital radiographs submitted for hip dysplasia screening. Front Vet Sci. (2019) 6:428. 10.3389/fvets.2019.00428 31850383 PMC6901622

[7] Suzuki A Matsubara K Chusin T Sasa Y. Eye lens doses of radiology technologists who assist patients during radiography. Radiat Prot Dosimetry. (2019) 185:275–81. 10.1093/rpd/ncz007 30753707

[8] Veterinary Practice News (2012). Available online at: https://www.veterinarypracticenews.com/when-it-comes-to-radiation-alara-can-save-your-life/ (accessed March 8, 2021).

[9] Elshamia W Abuzaida M Rajab O Almajed N Alnuwaiser O Alghareeb A. A snapshot of occupational radiation dose in veterinary radiology. Radiat Phys Chem. (2020) 168:108581. 10.1016/j.radphyschem.2019.108581

[10] Thrall DE. Textbook of Veterinary Diagnostic Radiology. St. Louis, MO: Elsevier (2007). p. 832.

[11] Miljø-og Fødevareministeriet. Bekendtgørelse af lov om dyreforsøg [Internet]. (2014). Available online at: https://www.retsinformation.dk/eli/lta/2014/474 (accessed March 9, 2021).

[12] University of Southern Denmark (2019). Available online at: https://sdunet.dk/en/vaerktoejer/love_regler_aftaler/forskning/etiske+retningslinjer+anvendelse+af+dyr (accessed March 9, 2021).

[13] Precht H Hansson J Outzen C Hogg P Tingberg A. Radiographers' perspectives' on Visual Grading Analysis as a scientific method to evaluate image quality. Radiography. (2019) 25(Suppl. 1):S14–8. 10.1016/j.radi.2019.06.006 31481182

[14] Håkansson M Svensson S Asplund S Svalkvist A Båth M Månsson L. ViewDEX 2.0: A Java-Based DICOM-Compatible Software for Observer Performance Studies. Bellingham, WA: Proc SPIE.

[15] McGraw KO Wong SP. Forming inferences about some intraclass correlation coefficients. Psychol Methods. (1996) l:30–46. 10.1037/1082-989X.1.1.30

[16] Meisinger QC Stahl CM Andre MP Kinney TB Newton IG. Radiation protection for the fluoroscopy operator and staff. AJR Am J Roentgenol. (2016) 207:745–54. 10.2214/AJR.16.16556 27440524

[17] Santos JS Uusi-Simola J Kaasalainen T Aho P Venermo M. Radiation doses to staff in a hybrid operating room: an anthropomorphic phantom study with active electronic dosimeters. Eur J Vasc Endovasc Surg. (2020) 59:654–60. 10.1016/j.ejvs.2020.01.018 32061447

[18] Koernig K. The effect of collimation on image quality and radiation safety in digital radiography of small animals (PhD thesis). Auburn University, Auburn, AL, United States (2013). Available online at: https://etd.auburn.edu/xmlui/bitstream/handle/10415/3720/koernig%20thesis2.pdf?sequence=4&isAllowed=y (assessed March 19, 2021).

[19] Precht H Mørup S Tingberg A Outzen C Kusk K Nielsen R. Can scattter correction software replace a grid in dr pelvic examinations? Radiat Prot Dosimetry. (2019) 187:8–16. 10.1093/rpd/ncz129 31111927

[20] Karami V Zabihzadeh M. Beam Collimation during Lumbar Spine Radiography: a retrospective study. J Biomed Phys Eng. (2017) 7:101–6. 28580331 PMC5447246

[21] Pazanin A Skrk D O'Driscoll JC McEntee MF Mekis N. Optimal collimation significantly improves lumbar spine radiography. Radiat Prot Dosimetry. (2020) 189:420–7. 10.1093/rpd/ncaa057 32363403

[22] Mc Fadden S Roding T de Vries G Benwell M Bijwaard H Scheurleer J. Digital imaging and radiographic practise in diagnostic radiography: an overview of current knowledge and practice in Europe. Radiography. (2018) 24:137–41. 10.1016/j.radi.2017.11.004 29605110

[23] Seeram E Davidson R Bushong S Swan H. Optimizing the exposure indicator as a dose management strategy in computed radiography. Radiol Technol. (2016) 87:380–91. 26952062

[24] Santana A Alves-Pimenta S Martins J Colaco B Ginja M. Hands-free conventional radiographic ventrodorsal hip extended view. Front Vet Sci. (2020) 7:286. 10.3389/fvets.2020.00286 32587864 PMC7297906

